# Desmoplastic Small Round Cell Tumour of Childhood: A Report of Four Cases Demonstrating Wider Clinical Features and Variable Outcome

**DOI:** 10.1080/13577149778380

**Published:** 1997-06

**Authors:** Ruma Ray, Heidi C. L. Traunecker, Faro Raafat, Michael C. G. Stevens

**Affiliations:** 1 Department of Histopathology Birmingham Children's Hospital UK; 2 Department of Oncology Birmingham Children's Hospital NHS Trust Birmingham B16 8ET UK

## Abstract

*Purpose*. Four further cases of desmoplastic small round cell tumour with multi phenotypic differentiation are described.

*Subjects*. Two patients were typical (male, adolescent with peritoneal tumour and, in one case, liver metastases) and did not respond well to treatment. Two other patients showed less usual features (young, female with retroperitoneal disease, both with intraspinal extension and renal tract obstruction). Both responded favourably to multi-modal treatment regimens including extensive and invasive supportive care.

*Results*. Histologically, all tumours showed clear features of this diagnosis, namely angulated nests of small cells in a background of fibrovascular stroma. Immunohistochemistry typically showed divergent differentiation with neural, muscle and epithelial marker positivity. All four tumours stained positive for the Wilms' tumour 1 suppressor gene product. Electron microscopy showed intercellular tight junctions, cytoplasmatic intermediate filaments and absence of microvilli. Rare neurosecretory-type granules were observed.

*Discussion*. These cases illustrate a broader spectrum of clinical presentation than previously associated with this diagnosis.


*Sarcoma* (1997) **1**, 103–108

CASE REPORT

### Desmoplastic small round cell tumour of childhood: a report of four cases demonstrating wider clinical features and variable outcome

RUMA RAY,[1] HEIDI C. L. TRAUNECKER,[2] FARO RAAFAT[1]
& MICHAEL C. G. STEVENS[2]

*Departments of [1]Histopathology and [2]Oncology, Birmingham Children's Hospital, UK*

**Abstract**

*Purpose.* Four further cases of desmoplastic small round cell tumour with multi phenotypic differentiation are described.
*Subjects.* Two patients were typical (male, adolescent with peritoneal tumour and, in one case, liver metastases) and did not respond well to treatment. Two other patients showed less usual features (young, female with retroperitoneal disease, both with intraspinal extension and renal tract obstruction). Both responded favourably to multi-modal treatment regimens including extensive and invasive supportive care.
*Results.* Histologically, all tumours showed clear features of this diagnosis, namely angulated nests of small cells in a background of fibrovascular stroma. Immunohistochemistry typically showed divergent differentiation with neural, muscle and epithelial marker positivity. All four tumours stained positive for the Wilms' tumour 1 suppressor gene product. Electron microscopy showed intercellular tight junctions, cytoplasmatic intermediate filaments and absence of microvilli. Rare neurosecretory-type granules were observed.
*Discussion.* These cases illustrate a broader spectrum of clinical presentation than previously associated with this diagnosis.

**Key words**: *desmoplastic small round cell tumour, clinical presentation.*

### Introduction

The so-called 'malignant intra-abdominal small round cell tumour with desmoplastic reaction' is a relatively recent addition to the group of small blue round cell tumours of childhood.[1] Its clinical presentation is characterized by its adolescent male predominance and by its rare occurrence outside the peritoneal cavity. Pathologically, these tumours show two distinct elements—a population of small malignant cells which demonstrate multi phenotypic characteristics, and a benign spindle cell stroma. The prognosis is usually poor and the majority of patients with such tumours reported previously have shown only partial response to chemotherapy or radiotherapy.[2–4] There have, however, been reports of a wider clinical presentation with involvement of other intra-abdominal or serosal surfaces including ovaries, scrotum and pleura, and at distant sites including the central nervous system (CNS).[5–8]

We present four further cases of this rare tumour which contribute to the recognition of its wider clinical manifestations. Two patients with typical presentation (adolescent males with peritoneal disease) did badly but two others, both young girls with retroperitoneal tumours, have shown a more encouraging response to treatment.

### Subjects and methods

All four cases presented to Birmingham Children's Hospital for investigation of an abdominal mass. The diagnosis was made utilizing computed tomography (CT)-guided tru-cut needle biopsy in three cases and by primary excision of the entire abdominal mass in the fourth patient. Haematoxylin and eosin (H&E)-stained sections of formalin-fixed, paraffin-embedded tissue were examined from all four patients. A wide range of immunohistochemical stains were applied using an avidin–biotin–peroxidase technique (Table 1). In addition, special stains for glycogen (periodic acid schiff (PAS), with and without diastase), reticulin, trichome and the Wilms' tumour 1 (WT1) gene product[9] were used in all cases. Electron microscopy was carried out on fresh tissue fixed in buffered glutaraldehyde. The sections were stained with uranyl acetate and lead citrate and examined with a Zeiss electron micro-

Correspondence to: M. C. G. Stevens, Department of Oncology, Birmingham Children's Hospital NHS Trust, Birmingham B16 8ET, UK. Tel: +44 121 450 6153; Fax: +44 121 450 6441; E-mail: mike.stevens@bhamchildrens.wmids.nhs.uk.

1357-714X/97/020103-06 $9.00 ©1997 Carfax Publishing Ltd

**Table 1.** *Immunohistochemical panel*

| Antibiotics | Source | Dilutions |
|---|---|---|
| Desmin | DAKO M760 | 1:50 |
| Keratin | EURODIAGNOSTIC PKE | 1:40 |
| EMA (epithelial membrane antigen) | DAKO M613 | 1:40 |
| NSE (neuron-specific enolase) | DAKO M873 | 1:100 |
| S100 | DAKO Z331 | 1:500 |
| EWINGS HBA 71 | Signet 013 (P1146) | 1:40 |
| LCA (leucocyte common antigen) | DAKO M701 | 1:50 |
| Synaptophysin | DAKO A010 | 1:50 |
| SMA (smooth muscle actin) | Biogenex MU090-UC | 1:20 |
| WT1 (Wilms' tumour 1 gene product) | Santa Cruz Biotechnology | 1:600 |

scope. Cytogenetic analysis was unsuccessful in the one case for which satisfactory tissue was available.

### Clinical presentations

Clinical details of the four patients are summarized in Table 2. Three patients presented with symptomatic, extensive pelvic masses of which two extended into the spinal canal (cases 3 and 4). Radiological examination with CT scan proved helpful in visualizing the extent of disease at diagnosis including the intraspinal extension, presence of metastases (multiple liver metastases in case 1) and involvement of the renal tract, although it subsequently failed to identify progressive peritoneal seeding in case 1. Patient 3 presented with bilateral severe hydronephrosis due to obstruction of both ureters by the tumour mass and required insertion of bilateral nephrostomies prior to chemotherapy and treatment for renal hypertension. Case 4 presented with hydronephrosis due to ureteric obstruction in a horseshoe kidney, complicated by hypertension.

One patient (case 2) who presented with re-

**Table 2.** *Patient characteristics*

| Case | 1 | 2 | 3 | 4 |
|---|---|---|---|---|
| Age (years)/Sex | 15/M | 11/M | 5/F | 4/F |
| Presentation | Weight loss, malaise, abdominal mass, breathlessness on exertion | Vague intermittent upper abdominal pain | Painful limp, anorexia, constipation, urinary symptoms, hydronephrosis, hypertension, lymphoedema left leg | Abdominal pain, incomplete paraplegia, neuropathic bladder, hypertension, hydronephrotic horseshoe kidney |
| Site and extent of tumour at diagnosis | Large pelvic tumour, hepatic metastases and possible single bone metastasis | Single mobile tumour mass arising from omentum | Large pelvic retroperitoneal tumour, extension in spinal canal | Large pelvic retroperitoneal tumour, extension in spinal canal |
| Chemotherapy | * | ** | *** | *** |
| Surgery | Peritoneal metastases at second-look surgery | Primary resection of tumour | Oophoropexy prior to radiotherapy transuretero-ureterostomy | Oophoropexy prior to radiotherapy |
| Further treatment | (1) Multi-drug resistance modulation† (2) Oral etoposide | (1) Second-line chemotherapy†† (2) Oral etoposide | 45 Gy to pelvis and spinal canal | 45 Gy to pelvis and spine |
| Disease status | Alive with residual disease at 18 months | Died at 5 months from metastatic disease | Alive at 30 months without evidence of disease | Alive at 14 months without evidence of disease |

*Vincristine, ifosfamide, actinomycin D, etoposide, carboplatin, epirubicin.
**Vincristine, ifosfamide, actinomycin D.
***Vincristine, cyclophosphamide, actinomycin D.
†Etoposide, vincristine, epirubicin with cyclosporin A.
††Vincristine, carboplatin, etoposide.

current abdominal pain but was otherwise well underwent primary surgical resection of the intraperitoneal tumour at diagnosis and macroscopically complete resection was thought to have been achieved. No peritoneal seeding was found at surgery but microscopically the margins were not clear and within 3 months of commencing chemotherapy (using vincristine, ifosfamide and actinomycin D) the tumour recurred widely within the peritoneal cavity. Second-line chemotherapy and palliative treatment with oral etoposide were unsuccessful in controlling the recurrence and the patient died of disease 5 months after diagnosis. Patients 3 and 4 received the same initial chemotherapy (vincristine, cyclophosphamide and actinomycin D), to which both showed a very good response. Residual disease within the spinal canal was treated by radiotherapy. To avoid long-term sequelae due to pelvic radiotherapy, both patients underwent oophoropexy. Due to scarring of one ureter, resulting in persistent ureteric obstruction, patient 3 underwent left to right transuretero-ureterostomy. Patients 3 and 4 were alive and disease free at 30 and 14 months, respectively, following diagnosis.

The only patient (case 1) presenting with metastases (liver and a possible solitary bone metastasis) was treated with a metastatic sarcoma protocol to which he initially responded. Plans to resect the residual pelvic tumour were abandoned at second-look surgery after unsuspected peritoneal seeding was discovered throughout the abdominal cavity. A subsequent attempt to counteract presumed multi-drug resistance (MDR) utilizing cyclosporin A failed and further intensive treatment was refused. This patient was, however, alive 18 months after diagnosis with stable residual metastatic disease after palliative treatment with oral etoposide.

### Histopathology

### *Gross findings*

Three patients had tru-cut needle biopsies. The gross description was confined to the single patient (case 2) whose tumour was totally excised. This showed a solid and cystic mass weighing 438 g and irregularly lobulated. The tumour measured $13 \times 9 \times 7$ cm and appeared to be surrounded by a pseudocapsule.

### *Microscopic appearance*

The light microscopic appearances were identical in all four cases. These showed irregular islands of small, uniformly sized tumour cells with moderate amounts of pale cytoplasm with indistinct cell borders separated by a cellular fibrous stroma (Fig. 1). The tumour cell nuclei appeared slightly irregular with clumped chromatin and marked hyperchromasia. Nucleoli were prominent only in occasional cells. Mitoses and cell necrosis were frequent. Rare larger cells with hyalinized cytoplasm and eccentrically placed nuclei, resembling rhabdoid tumours, were identified. The spindle cell stroma separating the tumour nests had a benign appearance with no mitoses or necrotic areas.

### *Immunohistochemical findings*

These are summarized in Table 3. The tumour cells stained positively for vimentin and desmin. The latter showed the typical paranuclear dots (Fig. 2). Epithelial cell markers (keratin, epithelial membrane antigens (EMA)) as well as neural markers (NSE, S100) were positive in most cases. All four tumours stained positive for WT1 (Fig. 3). Ewing's sarcoma antibody (HBA71) was negative as were synaptophysin and leucocyte common antigen (LCA). Smooth muscle actin was positive in three cases. Glycogen represented by PAS positivity was demonstrated in most cells. The reticulin stain showed packeting of groups of cells and Masson trichrome stain showed well laid down collagen in the intervening stroma.

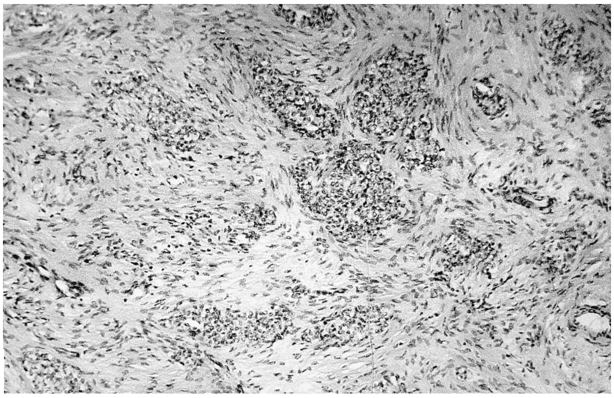

**Fig. 1.** *Well-defined clusters of tumour cells separated by dense stroma (H&E; ×78).*

**Table 3.** *Immunohistochemistry profile*

| Case | Keratin | EMA | Vimentin | Desmin | SMA | NSE | WT1 |
|---|---|---|---|---|---|---|---|
| 1 | + + + | + + + | + + | + + | + | + | + |
| 2 | + + + | + + + | + + | + + | − | + | + |
| 3 | + + + | + + + | + + | + + | + | + | + |
| 4 | + + | ND | + + + | − | + | − | + |

+ → + + + = weak → strongly positive.

− = negative.

ND = not done; SMA = smooth muscle actin; NSE = neuro-specific enolase.

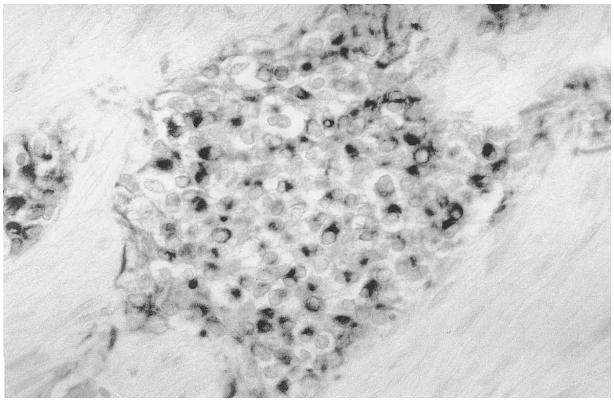

**Fig. 2.** *Desmin-positive tumour cells showing typical paranuclear 'dot-like' reaction (immunoperoxidase);* $\times$ *300.*

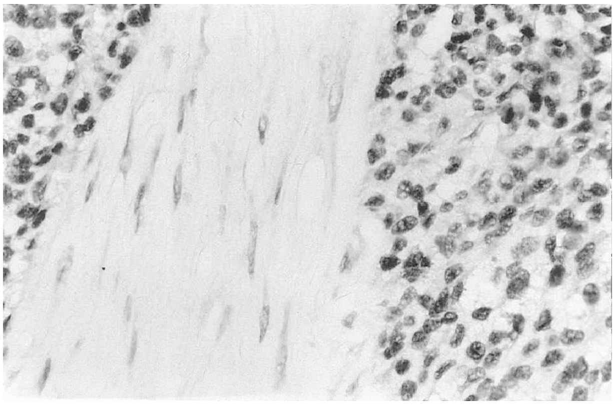

**Fig. 3.** *The nuclei in cellular and stromal areas of the tumour stain positive for WT1 (immunoperoxidase);* $\times$ *133.*

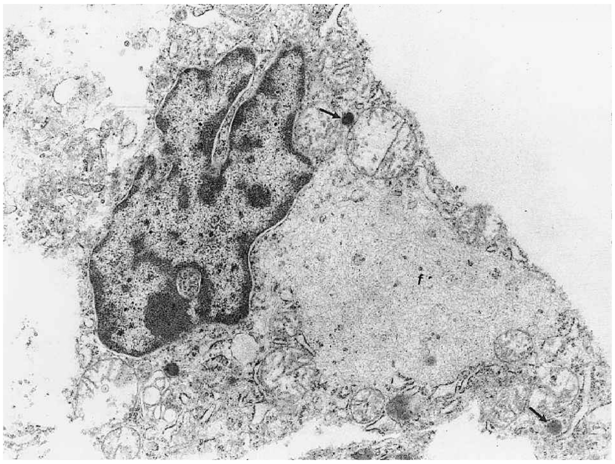

**Fig. 4.** *Paranuclear aggregate of intermediate filaments (f). Dense core granules indicated by arrows (*$\times$*6900).*

*Electron microscopic findings*

The tumour cells showed several tight junctions with occasional perinuclear intermediate-type filaments (Fig. 4). No lumens or microvilli were seen. Occasional cells showed dense core granules resembling neurosecretory-type granules. Rough endoplasmic reticulin was abundant in most cells. The nuclei had jagged irregular borders with occasional prominent nucleoli and marginated chromatin. External laminae were detectable focally.

### Discussion

The term intra-abdominal desmoplastic small cell tumour with divergent differentiation (DSRCT) was first used by Gerald and Rosai in 1989 to describe a case report of an abdominal tumour in an 8-year-old girl resembling other small blue round cells of childhood and showing epithelial, mesenchymal and neural differentiation.[1] Although they speculated that the tumour could have orginated from the peritoneal serosal layer, they could not prove its true histogenesis. Later, the same authors and their colleagues reported 18 additional cases and concluded that this was an aggressive group of undifferentiated small round tumours which, in contrast to the first case, were more predominant in males and commonly located within the abdomen.[2] Others have reported similar cases under the same or different rubric.[3,4,10]

This tumour has characteristic light microscopic features consisting of uniform cells with round to oval nucleus and clumped chromatin. Nucleoli are rare. The cytoplasm is of moderate amounts with indistinct cell borders. Mitotic figures and individual cell necrosis are common. Occasional cells show hyaline eosinophilic cytoplasm and displaced nuclei mimicking a malignant rhabdoid tumour. The glycogen, as demonstrated by PAS diastase-sensitive stain, is usually sparse. The reticulin stain delineates the clustering of groups of tumour cells separated by a dense fibrovascular stroma containing spindle-shaped cells and small calibre blood vessels. This desmoplastic feature is very characteristic and separates this group of tumours from other small blue round cell tumours of childhood. Immunohistochemically, these tumours can show positive reactions with antisera for epithelial (cytokeratin, EMA), mesenchymal (vimentin, desmin) and neural (NSE, S100) synaptophysin, and occasionally smooth muscle actin. Immunohistochemical staining for the WT1 gene product in desmoplastic small round cell tumours, which was positive in all four cases, has not been previously reported. The nuclei in cellular and stromal areas stained positive. Glial fibrillary acid protein and other markers for neurofilaments have been persistently negative, as have myoglobin and chromogranin.[11] The intensity of staining is usually greatest with epithelial cell markers followed by mesenchymal and finally neural markers. Electron microscopically, these tumours show features of malignant epithelial tumours with tight junctions and desmosome-like attachments between cells.[5,10,12,13] Neurosecretory-like granules, fat droplets and perinuclear collections of intermediate-type filaments are variably described.[5,11,13] The presence of the latter is accounted for by 'dot-like' positivity of desmin in immunohistochemical stains. Basal lamina intracellular lumens with microvilli suggesting mesothelial derivation have been seen on occasions.[5,13]

DNA content of these tumours has been investigated by some investigators.[13,14] Most cases showed hyperdiploidy with high S-phase proportions and proliferation indices suggestive of an aggressive nature. Cytogenetic analysis of these tumours has identified an association with a translocation between chromosomes 11 and 22 [t(11;22) (pl3;ql2)] but although this involves the 22ql2 breakpoint identified in the translocations site found in Ewing's sarcoma/primitive neuroectodermal tumour, the translocation site on chromosome 11 (llpl3) is different. In fact, this translocation shows a fusion of the Ewing's sarcoma (EWS) gene and the WT1 gene.[15,16] This finding raises further interest in the cell of origin of this tumour and in particular in the role of WT1 (a gene expressed in mature mesothelial cells) in the pathogenesis of DSRCT. The availability of an immunohistochemical technique, applicable in formalin-fixed tissue, for the WT1 gene product[9] allows further investigation into the expression of the WT1 gene product, particularly in light of the difficulty with which cytogenetic analysis of tumour material can be achieved.

Two particular clinical aspects of this tumour have been their predilection for peritoneal sites and their predominance in boys. The former had led to the original authors to qualify the diagnosis with the appellation of 'intra-abdominal' although the two retroperitoneal cases in our series and a recent report of this tumour at other sites[5–8] emphasize the necessity of keeping a broader view regarding the site and histogenesis of these tumours. However, the hypothesis that they originate in the mesothelial serosal layer is strengthened by the reports of their presence in the pleura. Furthermore, the ultrastructural presence of microvilli and intracellular lumens (the features of mesothelial neoplasms) reported by some authors supports the possibility of a mesothelial origin.[5,13] The immunohistochemistry does not refute this theory as, with the possible exception of desmin (a skeletal and smooth muscle cell marker), all other markers reactive in DSRCT could also be positive in tumours of mesothelial origin. Cases of this tumour have been reported in the extra-abdominal peritoneum (tunica vaginalis),[6,13,17] which strongly suggests the serosal surface as the most likely site of origin. A report of three cases of ovarian involvement by this tumour[5] could also be accounted for by the fact that the ovaries are covered by serosal surface.

The second original observation that this tumour is seen almost exclusively in males (usually of adolescent age) is not explained, although further publications including this have reported DSRCT in young girls. Experience implies that these tumours convey a poor prognosis,[2] but two of our four cases (both young females) showed a prompt response to initial chemotherapy and subsequently achieved complete remission, supporting the need for aggressive supportive care. Three of the four cases were alive at 14, 18 and 30 months beyond diagnosis although one (case 1) had stable residual disease. The patient who died within 5 months of diagnosis surprisingly presented with low tumour burden and limited disease, which behaved very aggressively. It is clear that this diagnosis may represent a broader spectrum of disease than first described although optimal therapy remains uncertain.

**Acknowledgements**

The authors wish to thank Mrs C. Evans for her assistance with immunohistochemistry, Mr D Redfern for electron microscopy and Mrs J. Clarke and Miss B. H. Jackson for typing the manuscript.

**References**

1 Gerald WL, Rosai J. Desmoplastic small cell tumour with divergent differentiation. *Paediatr Pathol* 1989; 9:177–83.
2 Gerald WL, Miller HK, Battifora H, *et al.* Intra abdominal desmoplastic small round cell tumour. Report of 19 cases of a distinctive type of polyphenotypic malignancy affecting young individuals. *Am. J Surg Pathol* 1991; 15:499–513.
3 Gonzales-Crussi F, Crawford SE, Sun-chem Chi J. Intra abdominal desmoplastic small cell tumours with divergent differentiation. Observation of three cases of childhood. *Am J Surg Pathol* 1990; 14:633–42.
4 Porter JC, Conrad K. Desmoplastic soft tissue tumour. *Med Pediatr Oncol* 1992; 20:362–6.
5 Young RH, Eichhorn JH, Dickersin GR, *et al.* Ovarian involvement by the intra abdominal desmoplastic small round cell tumour with divergent differentiation: a report of 3 cases. *Hum Pathol* 1992; 23:454–64.
6 Seenherm I, Davis CJ, Mostofi FK. Undifferentiated malignant epithelial tumours involving serosal surfaces of the scrotum and abdomen in young males (abstract). *J Urol* 1987; 137:214A.
7 Parkash V, Gerald WL, Parma A, *et al.* Desmoplastic small round cell tumour of the pleura. *Am J Surg Pathol* 1995; 19:659–65.
8 Tison V, Cerasoli S, Marigi F, *et al.* Intracranial desmoplastic small cell tumour: report of a case. *Am J Surg Pathol* 1996; 20:112–17.
9 Ramani P, Cowell JK. The expression pattern of Wilms' tumour gene (WT1) product in normal tissues and paediatric renal tumours. *J Pathol* 1996; 179:162–8.
10 Variend S, Gerrard M, Norris PD, *et al.* Intra abdominal neuroectodermal tumour of childhood with divergent differentiation. *Histopathology* 1995; 18:45–51.
11 Cheung NYA, Khoo US, Chan KW. Intra abdominal desmoplastic small round cell tumour. *Histopathology* 1992; 20:531–4.
12 Lamovec J. Intra abdominal desmoplastic round cell tumour with expression of muscle specific actin. *Histopathology* 1994; 24:577–9.
13 Ordonez NG, El-Naggar A, Ro JY, *et al.* Intra abdominal desmoplastic small cell tumour: a light microscopic, immunocytochemical, ultra structural, and flow cytometric study. *Hum Pathol* 1993; 24:850–65.

14 Schmidt D, Koster E, Harms D. Intra abdominal desmoplastic small cell tumour with divergent differentiation: clinicopathological findings and DNA ploidy. *Med Pediatr Oncol* 1994; 22:97–102.

15 Sawyer JR, Tryka AF, Lewis JM. A novel reciprocal chromosome translocation t(11:22) (p13;q12) in intra abdominal desmoplastic small round cell tumour. *Am J Surg Pathol* 1992; 16:411–20.

16 Brodie SG, Stocker SJ, Wardlaw JC, *et al.* EWS and WT-1 gene fusion in desmoplastic small round cell tumor of the abdomen. *Hum Pathol* 1995; 26:1370–4.

17 Prat J, Matias-Guiu X, Algalia F. Desmoplastic small round cell tumour. *Am J Surg Pathol* 1992; 16:306–7.
